# Tuberculose multirésistante chez l'enfant: à propos de deux cas

**DOI:** 10.11604/pamj.2016.23.126.9041

**Published:** 2016-03-25

**Authors:** Hajar Slimani, Myriem Bricha, Fatima-Ezzahra Sqalli, Sanaa Hammi, Jamal-Eddine Bourkadi

**Affiliations:** 1 Service de Pneumo-Phtisiologie, Hôpital Moulay Youssef, CHU Rabat, Akkari, Faculté de Médecine et de Pharmacie de Rabat, 10000, Maroc; 2 Faculté de Médecine et de Pharmacie de Tanger, Maroc

**Keywords:** Tuberculose, multirésistante, enfant, Tuberculosis, multidrug-resistant, child

## Abstract

La tuberculose multirésistante chez l'enfant est une forme grave de la tuberculose, présentant un problème majeur de santé surtout dans les pays en voie de développement. Nous présentons le cas de deux enfants suivis dans notre formation pour tuberculose multirésistante mis sous schéma thérapeutique de deuxième ligne.

## Abstract (en)

Multidrug-resistant tuberculosis in children is a severe form of tuberculosis, which remains a major health problem especially in developing countries. We report the case of two children treated, during our training, for multidrug-resistant tuberculosis and put on second-line regimen.

## Introduction

La tuberculose multirésistante chez l'enfant est une forme grave de la tuberculose qui représente un problème majeur de santé du fait du contrôle difficile de la maladie et de l'absence des formes pédiatriques du médicament antituberculeux. Nous présentons le cas de deux enfants suivis dans notre formation pour tuberculose multirésistante.

## Patient et observation

### Observation 1

Il s'agit d'un enfant de deux ans, fils unique, bien vacciné selon le programme national d'immunisation, avec bon développement psychomoteur. Traité pour tuberculose pulmonaire à microscopie positive pendant 6 mois à l’âge de 18 mois, mis sous 2RHZ/4RH, la recherche des BK dans les crachats est demeurée positive au 6e mois invitant à réaliser un Genexpert dans les expectorations revenant positif avec résistance à la Rifampicine détectée. A l'admission dans notre établissement c’était un enfant en bon état général, avec un poids de 10 kg (-1DS), une taille de 76 cm (-1DS), cicatrice vaccinale présente, l'examen pleuropulmonaire ainsi que le reste de l'examen somatique était sans particularité. La radiographie thoracique de face a montré un infiltrat hilo-axillaire gauche excavé. Ainsi l'enfant a été mis sous Kanamycine, Ethionamide, Cyclosérine, Pyrazinamide, Ethambutol, Ciprofloxacine et Vit B6 avec bonne tolérance clinique. L’évolution a été marqué sur le plan clinique d'une prise de 3 kilos, avec sur le plan bactériologique une négativation à l'examen direct et à la culture dès le premier mois du traitement. Actuellement l'enfant est au 6e mois du traitement avec une bonne évolution clinique radiologique et bactériologique, ainsi on prévoit l'arrêt de la Kanamycine et continuer avec les autres médicaments pendant 18 mois.

### Observation 2

Il s'agit d'une patiente de 9 ans, traitée pour primo-infection tuberculeuse en 2013 par 2RHZ/4RH, elle a deux oncles décédés par tuberculose multirésistante confirmée par un test de sensibilité. A l'arrêt du traitement antibacillaire pour primo-infection tuberculeuse, la patiente a présenté une toux sèche associée à une fièvre non chiffrée et sueurs nocturnes profuses dans un contexte d'altération de l’état général invitant la réalisation de 3 recherche de BK dans les crachats revenues positives à l'examen direct et à la culture, le Genexpert a confirmé la résistance à la Rif et le test de Hain a objectivé une résistance à la Rifampicine et à l'Isoniazide. A l'admission c’était une patiente consciente, indice PS à 2, cachectique avec un poids de 21 kg, l'examen pleuropulmonaire ainsi que le reste de l'examen somatique étaient sans particularité. La radiographie thoracique de face à l'admission a montré de multiples opacités micronodulaires et nodulaires confluentes par endroit avec excavations prenant la totalité de l'hémithorax gauche et le tiers inférieur de l'hémithorax droit. La tomodensitométrie thoracique a objectivé un aspect en faveur de dilatation de bronches plus marquées à gauche et un syndrome alvéolo-interstitiel bilatéral avec adénopathies hilaires bilatérales, inter-bronchiques et médiastinales antérieures. Le bilan biologique a montré une anémie à 9g/dl hypochrome microcytaire avec fer sérique diminué et ferritinémie élevée, un syndrome inflammatoire biologique avec une VS accélérée à la première heure à 45 mm, une CRP à 48,5, le reste du bilan biologique était sans particularité. La décision était de mettre la patiente sous Kanamycine, Ethambutol, Pyrazinamide, Cyclosérine, Ethionamide, Ciprofloxacine, et Vit B6 avec bonne tolérance clinique. L’évolution a été marquée par un gain de 2 kg dès le premier mois du traitement, une légère amélioration radiologique du côté droit, tandis que sur le plan bactériologique les BK sont demeurés positifs au premier mois et se sont négativés au deuxième. La décision était de maintenir le traitement de la phase d'attaque pendant 8 mois.

## Discussion

La tuberculose multirésistante (MDR-TB) se réfère à la tuberculose maladie causée par des souches de Mycobactérium tuberculosis résistantes à l'isoniazide et la rifampicine. Les estimations de l'incidence de la maladie MDR-TB chez les enfants sont nécessaires pour comprendre l'ampleur de ce problème et faire en sorte que le traitement soit disponible, y compris les préparations de médicaments essentiels de la tuberculose pour enfants [1]. L'OMS estime 9,4 millions de cas de tuberculose dans le monde entier en 2008 dont 440 000 (3,6%) avait une tuberculose multirésistante. la tuberculose chez l'enfant est estimée quant à elle à 10-15% de la charge totale, mais la charge de la MDR-TB chez les enfants est peu connue avec des taux plus élevés dans les pays en développement [2]. Cela reflète le fait que les enfants sont plus susceptibles que les adultes d'avoir une maladie paucibacillaire et que les jeunes enfants (<5 ans) ne peuvent pas expectorer, empêchant ainsi le diagnostic microbiologique. Les jeunes enfants courent le plus de risque de maladie grave et de décès une fois infecté [1]. Selon l'OMS, les enfants doivent être suspectés d'avoir MDR-TB s'ils sont en contact avec un cas connu de MDR-TB ou ne répondent pas au régime antituberculeux standard [3]. Le diagnostic de la tuberculose chez les enfants repose souvent sur un ensemble d'arguments: une histoire de contact avec une source infectieuse de la tuberculose; des symptômes et des signes évocateurs de la tuberculose (par exemple toux chronique, perte de poids / retard de croissance ou des signes de tuberculose pulmonaire ou extra-pulmonaire); et des enquêtes spéciales (par exemple, test cutané tuberculinique (TST) et / ou la radiographie thoracique) [4]. La confirmation bactériologique de la maladie de la tuberculose résistante aux médicaments est plus difficile à atteindre chez les enfants que chez les adultes [1]. Le diagnostic de la résistance aux médicaments doit être fait par la culture et l'antibiogramme, mais la difficulté d'obtenir les sécrétions respiratoires, rend ainsi la moitié de tous les enfants ayant un diagnostic clinique de la tuberculose négatifs à l'examen direct et à la culture, ce qui rend la confirmation microbiologique difficile [1]. Les enfants atteints de MDR-TB sont gérés de la même façon que les adultes [4]. Les Directives de l'OMS recommandent le traitement jusqu’à 18 mois après la première culture négative (24 mois dans la XDR-TB). En fonction de la gravité de la maladie et les effets secondaires expérimentés, les agents doivent être administrés par voie parentérale pendant au moins 6 mois [3, 5]. Parce que les enfants sont souvent paucibacillaire, une courte durée du traitement (12 mois) peut être suffisante au début si la maladie est non extensive [6]. Une hospitalisation pour les 4-6 premiers mois du traitement est souvent nécessaire afin d'administrer les agents injectables de deuxième ligne et de surveiller les effets indésirables. Ces derniers sont plus fréquents que dans le traitement de première ligne; heureusement, ces effets indésirables peuvent généralement être gérés sans arrêt des médicaments [7].

## Conclusion

Chez tout enfant suspect de tuberculose, penser à la résistance en présence d'une source de contamination ou devant une rechute précoce ou un échec thérapeutique pour que la prise en charge thérapeutique soit la plus précoce possible.
